# Investigating the Potential of Ufasomes Laden with Nintedanib as an Optimized Targeted Lung Nanoparadigm for Accentuated Tackling of Idiopathic Pulmonary Fibrosis

**DOI:** 10.3390/ph17121605

**Published:** 2024-11-28

**Authors:** Heba M. Aboud, Adel A. Ali, Nada H. Mohammed, Ahmed H. E. Hassan, Eun Joo Roh, Shahira F. El Menshawe

**Affiliations:** 1 Department of Pharmaceutics and Industrial Pharmacy, Faculty of Pharmacy, Beni-Suef University, Beni-Suef 62514, Egypt; heba.aboud@pharm.bsu.edu.eg (H.M.A.); adel.ali@pharm.bsu.edu.eg (A.A.A.); shahira.elmenshawe@yahoo.com (S.F.E.M.); 2 Department of Pharmaceutics and Pharmaceutical Technology, Faculty of Pharmacy, Deraya University, Minia 61768, Egypt; 3 Department of Medicinal Chemistry, Faculty of Pharmacy, Mansoura University, Mansoura 35516, Egypt; 4 Chemical and Biological Integrative Research Center, Korea Institute of Science and Technology (KIST), Seoul 02792, Republic of Korea; 5 Division of Bio-Medical Science & Technology, University of Science and Technology, Daejeon 34113, Republic of Korea

**Keywords:** idiopathic pulmonary fibrosis, nintedanib, intratracheal inhalation, ufasomes, Box–Behnken design, pharmacokinetics

## Abstract

**Background/objectives**: Idiopathic pulmonary fibrosis (IPF) is a prevalent interstitial lung disease that typically progresses gradually, leading to respiratory failure and ultimately death. IPF can be treated with the tyrosine kinase inhibitor, nintedanib (NTD), owing to its anti-fibrotic properties, which ameliorate the impairment of lung function. This study aimed to formulate, optimize, and assess NTD-loaded ufasomes (NTD-UFSs) as a nanosystem for its pulmonary targeting to snowball the bioavailability and therapeutic efficacy of the drug. **Methods**: To investigate the influence of numerous factors on NTD-UFSs assembly and to determine the optimal formulation, Box–Behnken statistical design was implemented with the assistance of Design-Expert^®^ software. The thin-film hydration strategy was employed to fabricate NTD-UFSs. The optimum NTD-UFSs formulation was subsequently selected and subjected to additional evaluations. Also, using a rat model, a comparative pharmacokinetic analysis was scrutinized. **Results**: The optimal NTD-UFSs elicited an accumulative release of 65.57% after 24 h, an encapsulation efficiency of 62.51%, a zeta potential of −36.07 mV, and a vesicular size of 364.62 nm. In addition, it disclosed remarkable stability and a continuous cumulative release pattern. In vivo histopathological studies ascertained the tolerability of NTD-UFSs administered intratracheally. According to the pharmacokinetic studies, intratracheal NTD-UFSs administration manifested a significantly higher AUC_0–∞_ value than oral and intratracheal NTD suspensions, by approximately 5.66- and 3.53-fold, respectively. **Conclusions**: The findings of this study proposed that UFSs might be a promising nanoparadigm for the non-invasive pulmonary delivery of NTD.

## 1. Introduction

Idiopathic pulmonary fibrosis (IPF) triggers an irreversible decline in lung function by replacing healthy alveolar tissue with fibrous tissue [1,2]. Despite clinical trials of several medications, there are no effective preventative interventions due to a lack of knowledge of the pathogenetic pathways, particularly in IPF [3]. The most popular theory proposes that IPF is due to dysregulated healing after repeated microscopic injuries to the cells lining the airways [1]. It is believed that IPF, which mostly affects middle-aged and older adults, develops as a result of an abnormal repair response to injury to the alveolar epithelial cells. This response is marked by the secretion of excessive amounts of extracellular matrix components, which scar the lung, distort its architecture, and completely eliminate its ability to function [4].

Nintedanib (NTD) is an FDA-approved antifibrotic biopharmaceutical that has proved effectiveness at slowing the progression of IPF [5]. NTD is a triple tyrosine kinase inhibitor that blocks intracellular signaling pathways vital for the growth and survival of tumor tissues, as well as the proliferation, migration, and transformation of lung fibroblasts [1]. However, when administered orally, only a small NTD amount reaches the epithelial airway surface [6]. Consequently, high and frequent concentrations are necessary to achieve fruitful treatment, leading to serious systemic adverse effects [1]. Also, NTD is categorized as a BCS II medication possessing a diminished systemic bioavailability (4.7%) pursuant to its sparse aqueous solubility. In fact, the exploitation of oral treatment is frequently discontinued among patients with IPF due to diverse gastrointestinal adverse events that are commonly associated with it [7,8]. In this instance, the drug is preferred for inhaled administration due to its unique benefits, which include rapid clinical response, minimal systemic adverse effects, high local concentration, large sizeable alveolar surface area, circumvented first-pass hepatic metabolism, and a thin blood–alveolar barrier [9,10,11]. In addition, its painless and non-invasive nature ensures patient compliance, in stark contrast to other routes, such as parenteral administration [11]. This leads to a higher quality of life for patients and augmented therapeutic outcomes [1,10].

Developing a safe and effective inhaled medication requires selecting a suitable nano-cargo for administration and producing a respirable formulation alongside the pharmacologically active molecule. Several studies have examined the pulmonary administration of NTD delivery platforms, such as nanosuspension [6], liposomes [12], lipid polymer hybrid nanoparticles [13], niosomes [14], PLGA nanoparticles [15,16], and inhalable powders [1]. Recent studies have shown that the unsaturated fatty acid, oleic acid (OA), can entrap drugs and form ufasomes (UFSs). UFSs are bilayer vesicles that self-assemble into nanoscale structures; they are identified by the presence of hydrocarbon tails of fatty acids within the bilayer membrane and carboxyl moieties that come into contact with water [17,18]. The ionization of around half of the carboxyl groups occurs within a restricted pH range (7–9) required to produce UFSs [19]. Fatty acids generate oily particles when the pH falls below this threshold, while they yield soluble micelles when the pH rises above this threshold [20]. The penetration improvement of pharmaceuticals by OA is well-corroborated through various mechanisms [19]. OA has the potential to disrupt tight junctions, reduce mucous layer viscosity, and increase membrane fluidity [19,21]. Additionally, OA-based UFSs can create a depot, which facilitates the continuous release of active substances [22]. As far as we know, there has been no investigation into the potential harnessing of UFSs for the pulmonary delivery of NTD.

Consequently, this study aimed to elaborate NTD-UFSs that could be delivered via the pulmonic route to ensure the effective lung targeting of NTD. The Box–Behnken design (BBD) was utilized to achieve an effectively encapsulated and stabilized NTD formulation with desirable nanovesicular size and physicochemical properties. In addition, the optimal NTD-UFSs formulation was scrutinized for its irritating potential on rat lungs using an in vivo histopathological study. Furthermore, the pharmacokinetic profile of the intratracheal optimum NTD-UFSs formulation was compared to that of oral and intratracheal NTD suspensions in Wistar male rats.

## 2. Results and Discussion

### 2.1. Fabrication of NTD-UFSs

Substantial preliminary research was conducted to determine the most suitable conditions for preparing NTD-UFSs formulations. Since the primary goal of the NTD-UFSs formulation design was to include the lipid-soluble medication NTD in the ufasomal suspension for its pulmonary targeting, the aptitude of the tailored nano-cargo to entangle appreciable drug amounts with appropriate vesicular sizes is crucial. The surfactants tested were numerous, and the surfactant Span 60 exhibited the highest EE% when combined with cholesterol. Furthermore, the maximum EE% values and acceptable VS ranges were observed with OA, compared to various free fatty acids. Consequently, these findings were reassuring and prompting the selection of the three factors (X_1_: OA, X_2_: Span 60, and X_3_: cholesterol concentrations) to evaluate their impacts at different levels for optimizing the NTD-UFSs using the BBD. The film hydration technique was used to prepare the NTD-UFSs, and a transparent and continuous thin film was accomplished by utilizing a 10 mL mixture of methanol and chloroform. Table 1 shows the results for the response variables over 15 experimental runs with varying levels of independent factors. The extensive array of dependent factors indicated that the values of independent variables can substantially impact the features of UFSs. 

### 2.2. Experimental Design

**Figure 1:**
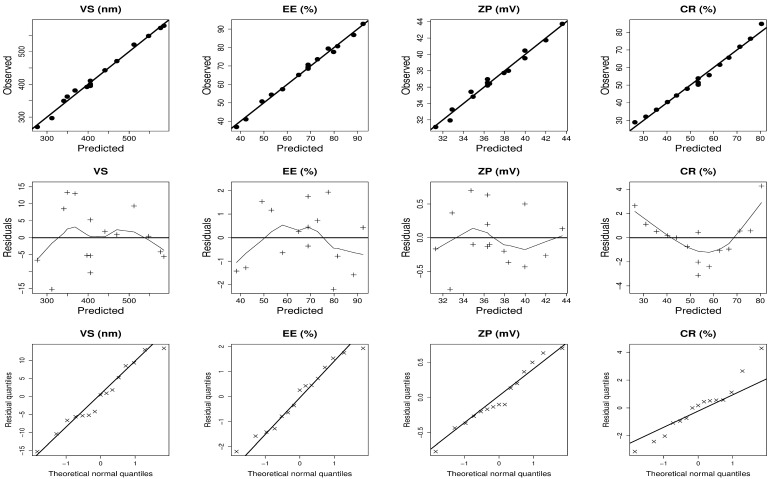
Model diagnostic plots for nintedanib-loaded ufasomes dependent variables. VS: vesicle size; EE%: encapsulation efficiency percent; ZP: zeta potential; CR: cumulative release percent following 24 h.

**Figure 2:**
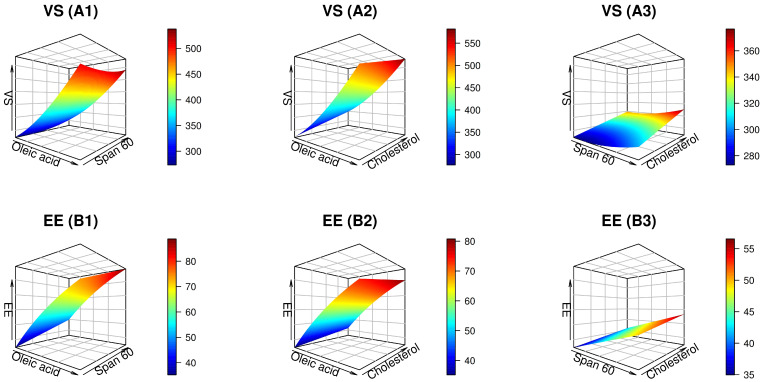
Three-dimensional plots showing the impact of independent factors on the vesicle size (VS) (**A1**–**A3**) and encapsulation efficiency (EE) (**B1**–**B3**) of nintedanib-loaded ufasomes.

**Figure 3:**
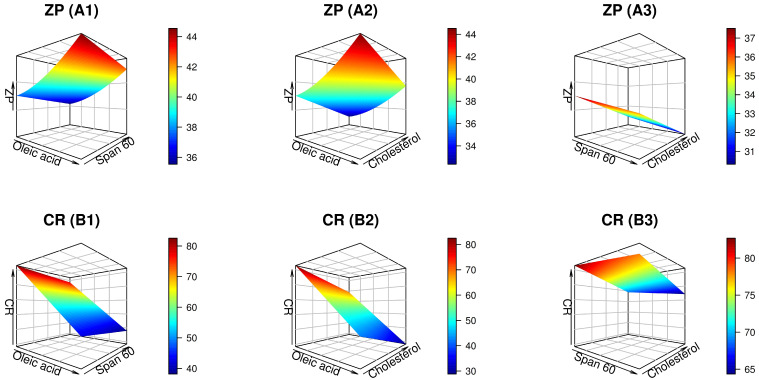
Three-dimensional plots showing the impact of independent factors on the zeta potential (ZP) (**A1**–**A3**) and cumulative release (CR) after 24 h (**B1**–**B3**) of nintedanib-loaded ufasomes.

Table 2 displays the design analysis values, wherein all responses showed that the adjusted R^2^ values agreed well with the predicted R^2^ values. The precision of all responses was greater than 4, as demonstrated in Table 2. The Design-Expert^®^ program used multiple regression analysis to conduct the statistical investigation. The model exhibiting the highest significant *p* value and insignificant lack of fit was chosen for representing each response. The magnitude and mathematical sign of each independent variable were assessed using regression equations. A positive sign indicates a synergistic impact, while a negative symbol denotes an antagonistic effect. Through a set of mathematical equations with coded values, Table 2 displays the fundamental relationships between each response and the independent factors. In addition, the ANOVA findings showed that all of the independent factors significantly affected each response. The diagnostic graphics in Figure 1 further demonstrated the adequacy of the fitted models. Graphical depictions in Figure 2 and Figure 3 manifest the correlation between each response and the causal factors. Figure 2 and Figure 3 provide three-dimensional (3D) plots that outline the relationship between two independent factors and each response, with the third variable being maintained at its central level.

#### 2.2.1. Analysis of VS

The lung deposition of nano-cargo is significantly influenced by the morphology and VS parameters. Table 1 and Figure 2(A1–A3) illustrate that the size of ufasomal formulations fluctuated from 269.83 ± 5.48 to 579.03 ± 5.25 nm. The enhanced diffusion mobility in this vesicular range makes it appropriate for better cellular absorption and effective medication administration into the lung [23]. The ANOVA demonstrated that all independent factors significantly influenced the VS of the generated UFSs formulation, *p* < 0.0001. Furthermore, the ANOVA findings for the VS data showed that the reduced quadratic model best fits the presented dataset. The VS was positively affected by all the three independent variables, as illustrated in Figure 2(A1–A3). Concerning OA levels, Figure 2(A1,A2) demonstrated that the ufasomal vesicles experience substantial growth as the formulation’s OA concentration increased (*p* < 0.0001). This expansion could be attributed to the increased viscosity that ensued from the raised free fatty acid content [24,25]. Pinilla et al. [26] shared parallel findings regarding enlarged nanovesicular size with OA integration during the elaboration of natural antimicrobial-based liposomes.

Additionally, the VS was detected to experience a substantial increase in response to the rise in Span 60 levels. Such observations emphasize the notion that surfactants having lengthier alkyl chains produce greater-sized nanovesicles. Since Span 60 is characterized by having an unsaturated long alkyl chain (C14), it was anticipated to yield larger UFSs [27]. Also, these outcomes match the EE% findings, as will be discussed later, which manifested a larger EE% of NTD with increasing the concentration of Span 60. As a consequence, the whole VS of NTD-UFSs was increased [23]. In addition, the low hydrophilic/lipophilic balance (HLB) of Span 60 (4.7) could be the reason behind these findings. Similar results were observed with azithromycin niosomes [28] and estradiol proniosomes [29].

Likewise, Figure 2(A2,A3) demonstrates that the production of larger vesicles directly resulted from an increase in cholesterol levels. The enlarged distance between UFSs bilayers was believed to result from the dense structure of cholesterol, which in turn provided a larger diameter size for NTD-UFSs [23,30]. Moreover, because of its unique geometrical packing attributes, cholesterol could mimic the hydrocarbon chains of amphiphilic surfactants. Besides, cholesterol might confer nanovesicles with limited water solubility and stiff-shaped membrane-stabilizing characteristics [31]. Subsequently, the utilization of elevated cholesterol levels in the UFSs formulation gave rise to the production of vesicles of larger sizes.

The PDI of the fabricated NTD-UFSs spanned from 0.211 ± 0.057 to 0.461 ± 0.056 (Table 1). Higher PDI estimates reflect greater heterogeneity, whereas lower values propose a limited size distribution with a uniform pattern of vesicular sizes [32]. In the current work, ANOVA testing for PDI values elucidated insignificant impacts of the three explored causal factors; hence, PDI appraisal was not enrolled in the optimization process.

#### 2.2.2. Analysis of EE%

High levels of drug trapping inside the vesicular structure are required to guarantee that a sufficient amount of the medication is delivered [33]. Consequently, it is imperative to mitigate the efflux of an entrapped drug substance within the nano-cargo. The data on EE% are depicted in a 3D surface diagram in Figure 2(B1–B3). As recorded in Table 1, the EE% of NTD-UFSs varied from 36.89 ± 2.18 to 92.83 ± 4.38%. The ANOVA results demonstrated that the EE% data were best suited by the reduced quadratic model. Furthermore, the ANOVA of the dependent factors showed that all causative elements significantly influenced NTD retention in UFSs, as compiled in Table 2, *p* < 0.0001.

The investigation results revealed that OA had a marked effect on EE% values, *p* < 0.0001. As depicted in Figure 2(B1,B2), this connection was directly proportional, meaning that an increase in OA produced a rise in EE%. The presence of a long alkyl chain in OA (C18) might cause this observation [33]. Additionally, OA improves the fluidity and flexibility of the lipid bilayer. Its hydrophobic tail and unsaturated fatty acid structure facilitate the improved packing and accommodation of drug molecules within the bilayer. This boosted encapsulation can be achieved by increasing the fluidity with more relaxed packing, creating more space for drug molecules [34]. Our results are congruent with those of Gabr et al. [35], who naratted higher EE% values (enriched from 63 up to 92%) of rosuvastatin-laden liquid crystalline nano-cargo upon incorporation of OA within the lipidic moiety.

Based on Figure 2(B1,B3), it is evident that the EE% of NTD experienced a synchronous upgrade as the concentration of Span 60 increased. The substantial drug encapsulation of Span 60, which results from its high phase-transition temperature of 53 °C, might help to explain this effect. The longer saturated hydrocarbon chain of Span 60 might also be a putative clarifier that generates robustly stable UFSs bilayers, thus enriching the EE% [36]. Moreover, Span 60 exhibits higher hydrophobicity, provoking a consolidated EE% owed to its lower HLB value [23]. Parallel observations were previously reported for olanzapine-based niosomes, which divulged a pronounced EE% when their Span 60 content was higher [37].

Notably, the EE% of NTD manifested a substantial increment as the cholesterol content in the NTD-UFSs formulations gradually upraised (*p* < 0.0001), as illustrated in Figure 2(B2,B3). As the cholesterol levels rose, the bilayers’ hydrophobicity and stability improved, while their permeability declined; this allowed for the effective formation of vesicles, which fruitfully trapped the hydrophobic drug within the vesicular bilayers. Such results are in line with those of Ahmed et al. [38], who scrutinized the potential of corneal-targeted novasomes loaded with fenticonazole nitrate for treating ocular candidiasis.

#### 2.2.3. Analysis of ZP

The stability of colloidal dispersions is predicted using ZP measurements; thus, it was considered during the optimization procedure [28,39]. The value of ZP is a measure of the magnitude of electrostatic repulsion triggered via nearby particles with identical charges and distributions. Hence, formulations with a greater ZP (negative or positive) elicit stability by reducing the generation of floccules and particle aggregation. Typically, formulations are considered stable if their surface charges are greater than +30 mV or less than −30 mV [40]. The ZP values of the produced UFSs varied between −31.14 ± 2.18 and −43.27 ± 3.43 mV, as shown in Table 1 and Figure 3(A1–A3), suggesting that the UFSs had favorable colloidal stability. According to the present results, all the tailored ufasomal dispersions were negative; thus, the manipulation of ZP data was pursued with regard to the absolute values to avoid misinterpretation [41]. The reduced quadratic model was found adequate for analysis based on ANOVA of the observed ZP data. Moreover, there was a statistically significant effect of each independent variable on the ZP of the UFSs formulations (*p* < 0.0001).

As anticipated, the values of ZP rose proportionately to the upsurge in OA content. The presence of its negatively charged carboxyl group may be the impetus of this phenomenon [42]. A negatively charged carboxylate moiety was produced when the carboxyl group dissociated in the lipid bilayer of UFSs when OA was incorporated.

On the contrary, the ZP and Span 60 levels exhibited a negative correlation, as illustrated in Figure 3(A1,A3). The absence of a net charge in Span 60 (sorbitan monostearate), a non-ionic surfactant, could elucidate this finding. Incorporating Span 60 into the lipid bilayer can mitigate the effects of negatively charged lipids, such as OA, and reorganize the lipid bilayer, potentially shielding or neutralizing the negative charges. This could decline the overall negative surface charge and diminish negative ZP.

Similarly, an elevated cholesterol level provoked formulations with low ZP values. Cholesterol may reduce the negative ZP of UFSs by intercalating into the lipid bilayer, which impacts the membrane’s fluidity and organization. Additionally, cholesterol molecules can mitigate or conceal the negative charges on the surface of the vesicles. This results in curtailing the negative ZP and the overall surface charge density. Furthermore, the bilayer structure is stabilized by the presence of cholesterol, which results in a more neutral-charge environment on the vesicular corona.

#### 2.2.4. Analysis of CR

Table 2 outlines how the independent elements affect NTD release from UFSs. Figure 3(B1–B3) shows the data plotted in a 3D surface plot. As indicated in Table 1, the CR% values oscillated from 28.90 ± 2.36 to 84.79 ± 2.17%. As noted from the ANOVA results, the CR data were best suited to the quadratic model and significantly impacted by the three independent factors (*p* < 0.0001).

As illustrated in Figure 3(B1,B2), the negative correlation between the CR of NTD and the quantity of OA incorporated can be explained by the alkyl chain of OA. The nanovesicles were less permeable due to the long, compact, and linear alkyl carbon chain of the OA. Therefore, it is reasonable to anticipate that the release of NTD from the ufasomal nano-cargo, which contained a high concentration of OA, to be delayed. Additionally, as already interpreted for the VS estimation, the increment of OA levels within the UFSs would yield larger-sized nanovesicles conferring a constricted surface area disclosable to the release medium, thereby curtailing NTD efflux.

Of note, a negative correlation between Span 60 levels and CR was revealed by the results depicted in Figure 3(B1,B3). The Span 60 solid-state composition, hydrophobicity, alkyl chain, and high phase-transition temperature could explain the diminutive drug release [43]. Prakash et al. [44] declared that the release of candesartan-loaded niosomes dropped from 100 to 77% when the Span 60 level was raised from 2.5 to 15 mg.

Upon further examination of the results, it was evident that the CR of NTD from the assembled ufasomal formulations was significantly influenced by cholesterol content, which had antagonistic consequences. In fact, the presence of cholesterol resulted in a more rigid and less permeable membrane, which enhanced the stability of the vesicles, provoking a sluggish impact on the drug release rates [23].

Based on the linear-regression investigation, the compiled release data revealed that the NTD efflux from the majority of the designed nanodispersions followed Higuchi kinetics, indicating a diffusion-monitored mechanism. Our findings are in agreement with diverse previous arts for drug-based nanovesicles [41].

### 2.3. Formulation Optimization

**Figure 4:**
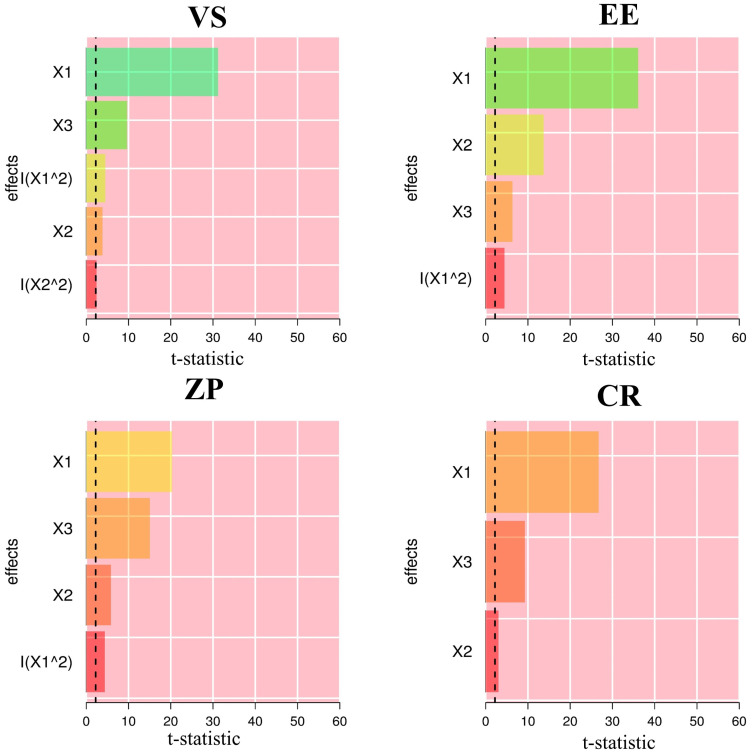
Pareto chart representing the standardized impacts of X_1_: oleic acid concentration (mg), X_2_: Span 60 concentration (mg), and X_3_: cholesterol concentration (mg). VS: vesicle size; EE: encapsulation efficiency; ZP: zeta potential; CR: cumulative release percent following 24 h.

The Design-Expert^®^ program was applied to determine the optimal formulation. The circumstances were optimal for generating nanovesicles with minimal VS, maximal ZP, EE, and CR. With a desirability index of 0.569, the optimum formulation contained 28.31 mg of OA, 129.28 mg of Span 60, and 20 mg of cholesterol, satisfying the UFSs formulation requirements. Table 3 shows the responses of the optimum formulation. The provided models effectively assessed the properties of the optimal UFSs. According to Table 3, the prediction error values for all responses were below 5%. As demonstrated in Figure 4, the Pareto chart clearly elucidates the persistent impact of formulation parameters on the response variables. All response variables were most significantly influenced by the levels of OA (X_1_).

### 2.4. Appraisal of the Optimal Formulation

#### 2.4.1. FTIR Analysis

**Figure 5:**
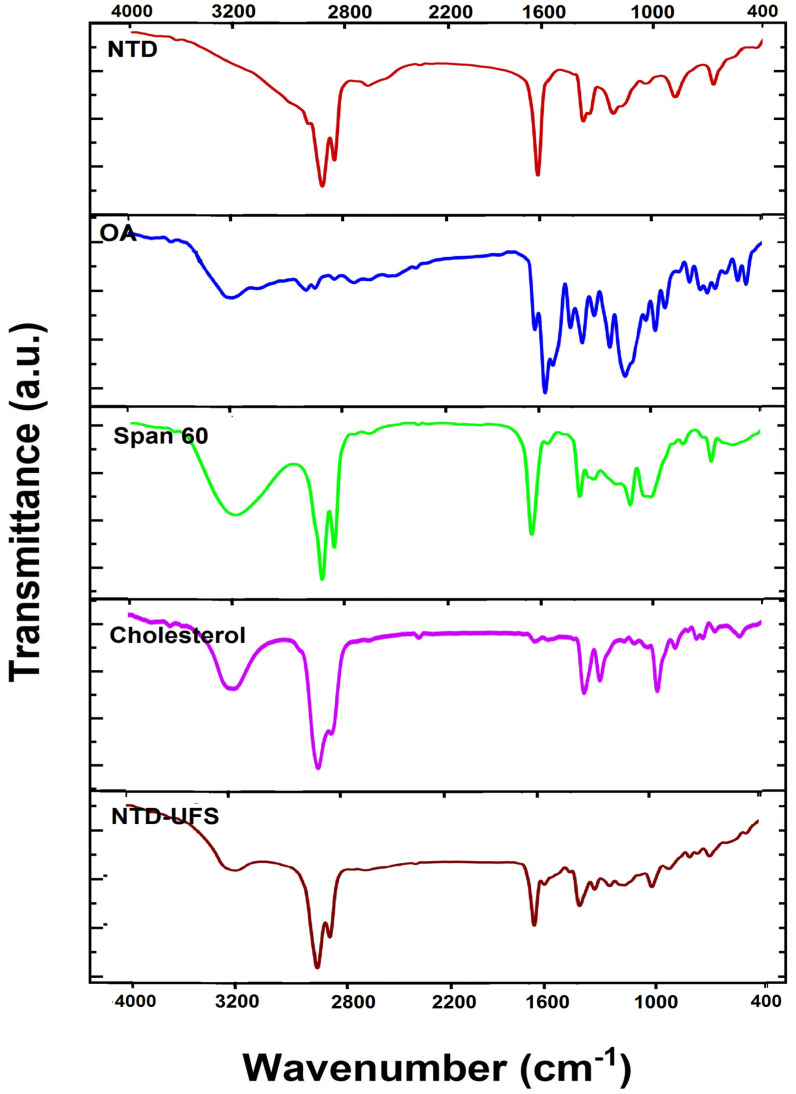
FT-IR spectra of nintedanib (NTD), oleic acid (OA), Span 60, cholesterol, and the optimum nintedanib-loaded ufasomes (NTD-UFSs).

Figure 5 displays the IR spectra of OA, Span 60, cholesterol, NTD, and NTD-UFSs (optimum formulation). The NTD spectrum manifests notable peaks at 2926.96, 2858.06 cm^−1^ (attributable to the C–H stretching of CH_3_), 2671.84, 2376.86, and 1712.08 cm^−1^ (representing the C=O stretching of the ester moiety), 1457.11 cm^−1^ (corresponding to the C=O stretching of the amide), 1422.14, and 1288.38 cm^−1^ (reflecting C–N stretching) [45]. There are three more noteworthy peaks at 1106.96, 939.52, and 723.83 cm^−1^. Furthermore, Figure 5 illustrates the IR spectra of cholesterol, Span 60, and OA. The IR spectrum of NTD-UFSs exhibited identical characteristic bands of NTD in the correspondent areas and magnitudes (Figure 5). It is obvious from these results that none of the components under investigation elicited any signs of chemical interactions with NTD [10].

#### 2.4.2. Differential Scanning Calorimetry (DSC)

**Figure 6:**
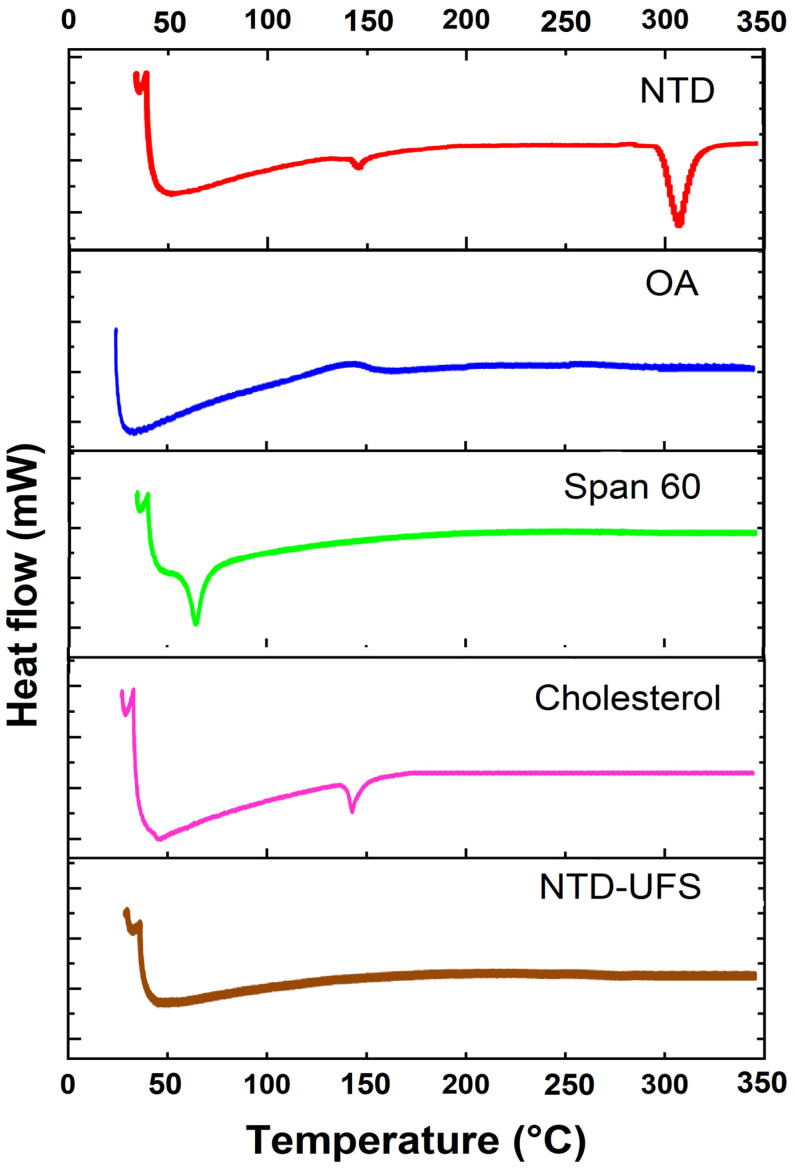
Differential scanning calorimetry thermograms of nintedanib (NTD), oleic acid (OA), Span 60, cholesterol, and the optimum nintedanib-loaded ufasomes (NTD-UFSs).

The influence of NTD inclusion and its degree of crystallinity within the nano-cargo was investigated by the thermal studies performed with the DSC instrument (Figure 6). The thermogram of NTD showed two notable endothermic peaks, which are at 143.95 °C (T_start_ = 140.50 °C; T_end_ = 146.37 °C; Heat = 13.72 J/g) and 308.32 °C (T_start_ = 295.26 °C; T_end_ = 316.48 °C; Heat = 53.86 J/g), consistent with previously documented findings in the literature on NTD [46]. The endothermic peaks emphasized the crystalline trait of NTD and that it was incorporated in its pure form. OA divulged a broad peak at 167.04 °C (T_start_ = 153.73 °C; T_end_ = 183.19 °C; Heat = 174.77 J/g). Span 60 manifested a sharp peak at 58.42 °C (T_start_ = 54.35 °C; T_end_ = 62.66 °C; Heat = 1090.70 J/g). Cholesterol exhibited an endothermic peak at 145.90 °C (T_start_ = 142.91 °C; T_end_ = 148.78 °C; Heat = 34.25 J/g). Nevertheless, the disappearance of NTD characteristic peaks in the NTD-UFSs thermogram proved the drug’s amorphous state, which is anticipated to snowball its permeability and solubility [47,48].

#### 2.4.3. In Vitro Release

**Figure 7:**
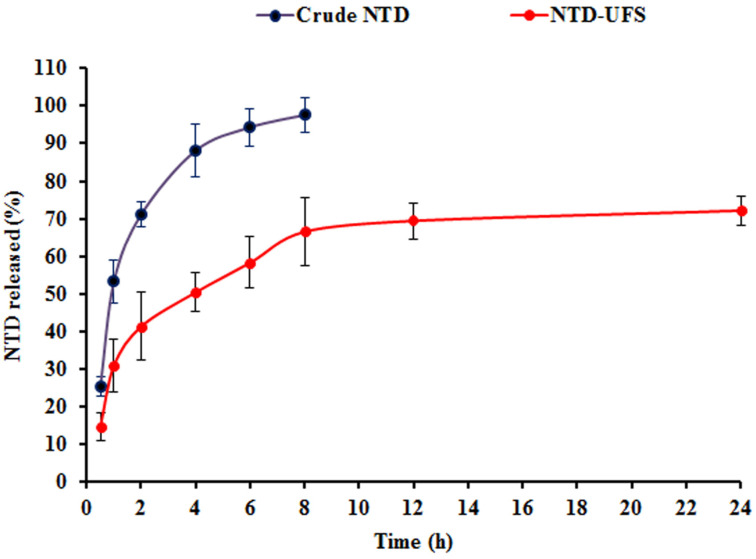
Mean accumulative nintedanib (NTD) percent released from the optimum nintedanib-loaded ufasomes (NTD-UFSs) formulation compared to crude nintedanib in phosphate buffered saline containing Tween 80 (0.5% *w*/*v*); mean ± SD; n = 3.

Utilizing the dialysis bag approach, the in vitro release profiles of crude NTD and the optimal NTD-UFSs dispersion were evaluated during 24 h, as shown in Figure 7. The crude NTD exhibited a cumulative release of 71.17 ± 5.86% during a 2 h duration, which then rose to 97.58 ± 2.16% after a total of 8 h. In contrast, the accumulative amount of NTD released from NTD-UFSs was 41.39 ± 4.92% during a 2 h timeframe and 72.19 ± 3.28% after 24 h. This might be owed to the notion that the drug is ensnared inside the UFSs nano-cargo, preventing it from diffusing unduly into the surrounding milieu. A biphasic pattern was noted for the in vitro release profile of NTD-UFSs, with a rapid burst release of the medication occurring within the first 2 h. Then, the drug’s release from the ufasomal dispersion was subsequently extended. The relative reservoir impact of the nanovesicular carrier sheds light on the possible explanation for the drug release from UFSs continuing prolonged following the first burst release.

#### 2.4.4. Morphological Analysis

**Figure 8:**
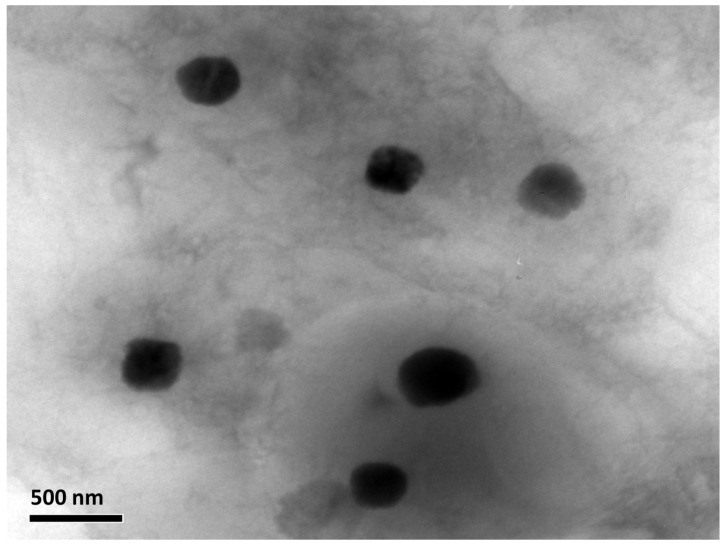
Transmission electron photomicrograph of the optimal nintedanib-loaded ufasomes.

Initially, TEM was implemented to investigate the morphology of the optimized NTD-UFSs formulation. The TEM image showed that the lipidic unilamellar nanovesicles had a homogenous size distribution and an approximately spherical shape (Figure 8). The vesicles were also well-separated and uniformly dispersed. The size analysis of the TEM micrographs also confirmed the nanosized range, which coincided with the DLS findings.

#### 2.4.5. Short-Term Stability

**Figure 9:**
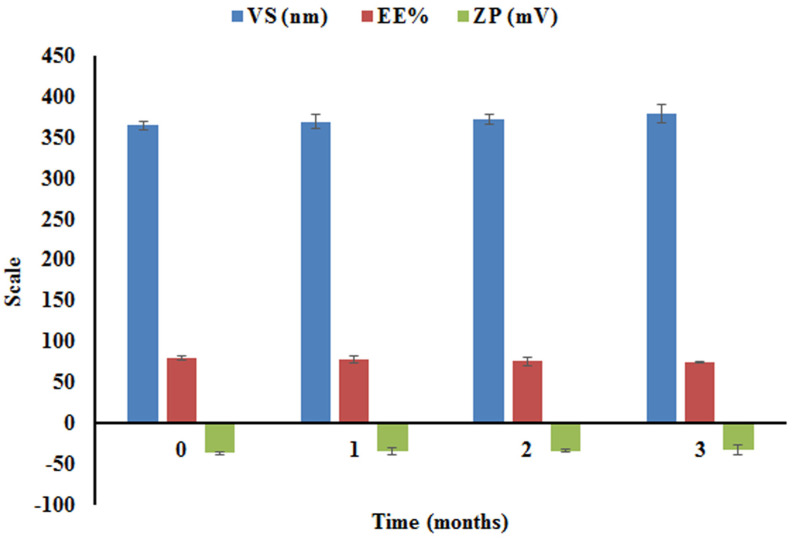
Effect of storage on the encapsulation efficiency percent (EE%), zeta potential (ZP), and vesicle size (VS) of the optimal nintedanib-loaded ufasomes formulation at 4 °C.

After being stored at 4 °C for three months, the optimum NTD-UFSs dispersion was evaluated for physical stability utilizing EE%, VS, and ZP values. Over the storage time, no aggregation or aberration was observed. Figure 9 elucidated that the EE%, VS, and ZP of the nanoformulation were not significantly affected by storage time. The dispersion stability was indicated by the highly negative ZP of the optimized NTD-UFSs formulation. It is possible that the coexistance of OA could trigger the net negative charges on the UFSs corona, which augmented the nano-cargo stability in the aqueous phase [23].

#### 2.4.6. Aerodynamic Characterization

A quality assurance procedure for an inhaler device can be established using in vitro methods to verify the inhalable released product’s quality. Also, such methods are commonly extrapolated to donate an estimate of the in vivo deposition of the inhaled formulation within the lungs. In order to ascertain the final deposition place, penetration depth, and accumulation inside the lung mucosal membranes, the particulate dimension of NTD-UFSs is essential [49]. According to the current findings, ACI stages 3–5 were the most sensitive for detecting the emitted NTDD-UFSs, which correspond to the lower airways and alveoli. Table 4 displays the aerodynamic characteristics of the tested formulation. A strong tendency for the deep deposition of the optimized NTD-UFSs into the lung tissue was indicated by the aerosol’s MMAD of 3.09 ± 0.06 µm and FPF of 83.61 ± 2.37%, which were released from the endotracheal tube. Thus, these findings suggested that the customized NTD-UFSs could exhibit strong pharmacological efficacy in lung disorders.

### 2.5. In Vivo Experimentations 

#### 2.5.1. Histopathological Analysis

**Figure 10:**
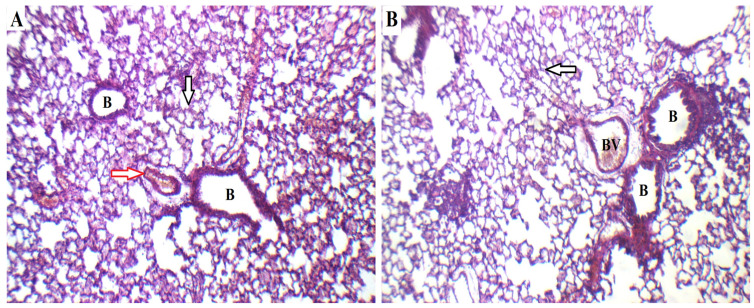
Histopathological images of the lungs of (**A**) normal control and (**B**) intratracheally administered optimum nintedanib-loaded ufasomes formulation. B: bronchioles; BV: blood vessels; Black arrow: alveolar walls; red arrow: blood vessels. (200× H & E).

To detect any acute toxicity of the applied nanoformulation, a histopathological analysis was performed by contrasting the lung tissues of rats exposed to the intratracheal optimized NTD-UFSs with those of normal rats. The inhalation technique may result in an asymmetrical distribution of drugs, so the lungs (right and left) were examined independently. Figure 10A represents the lung section of the normal control group, showing bronchioles (B), blood vessels (red arrow), and alveolar walls (black arrow). In addition, no swelling, epithelial damage, peribronchial, or perivascular inflammation was seen. Figure 10B clarifies the lung tissue of the optimum NTD-UFSs-treated group, revealing normal bronchioles (B), blood vessels (BV), and alveolar walls (black arrow). Furthermore, after receiving the NTD-UFSs formulation intratracheally, neither the right nor the left lung of the animals exhibited any significant tissue inflammation or damage. According to the findings of this investigation, the novel NTD-UFSs dispersion was biocompatible and did not produce acute toxicity due to its nanovector constituents.

#### 2.5.2. Pharmacokinetic Studies

**Figure 11:**
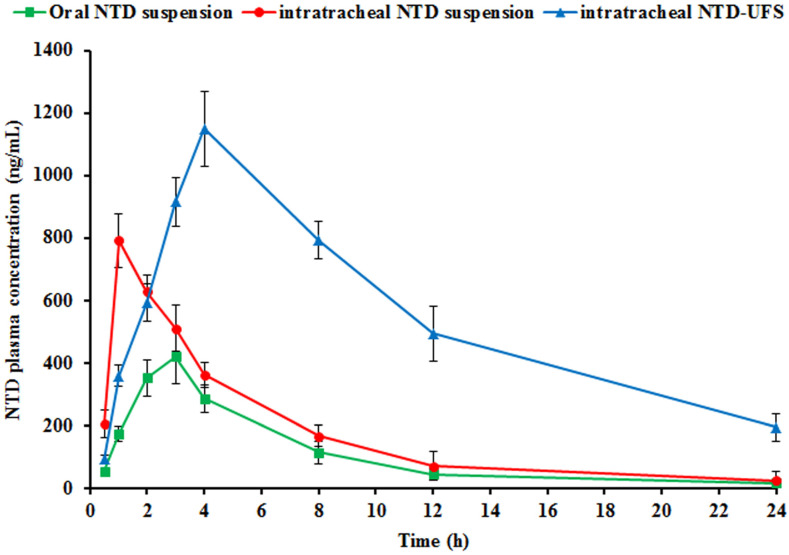
Nintedanib (NTD) levels in rat plasma following administration of oral NTD suspension, intratracheal NTD suspension, and intratracheal NTD-loaded ufasomes (NTD-UFSs).

Male Wistar rats received oral NTD suspension, intratracheal NTD suspension, and intratracheal NTD-UFSs suspension, and then their plasma concentrations of NTD were measured. Table 5 records the related pharmacokinetic parameters for different formulations, whereas Figure 11 reveals the mean plasma concentrations of NTD with time. Figure 11 outlines the plasma profile for intratracheal NTD-UFSs administration, which elucidates a slow increment in NTD concentration within 4 h supervened by an exponential fall in the circulation. Such findings could be ascribed to the extended release of NTD from the UFSs following administration. The in vitro release profile, as marked in Figure 7, also supports this phenomenon. The bloodstream was demonstrated to contain detectable levels of NTD for 24 h. This implies the continuous release of NTD from the developed NTD-UFSs.

According to the current findings, the T_max_ values were 1 and 3 h for the intratracheal and oral NTD suspensions, respectively. Nevertheless, as depicted in Figure 11, within less than 8 h, they were no longer in the circulation. Although the intratracheal NTD-UFSs suspension reached its T_max_ within 4 h, it remained detectable in the plasma for up to 24 h. The AUC_0–∞_ of NTD in plasma were 2690.06 ± 388.77, 4307.82 ± 159.76, and 15,221.19 ± 383.44 ng h/mL following administration of the oral NTD suspension, intratracheal NTD suspension, and intratracheal optimum NTD-UFSs formulation, respectively. It is crucial to observe that the AUC_0–∞_ of the intratracheal NTD-UFSs suspension was substantially higher than those of the oral and intratracheal NTD suspensions, with differences of approximately 5.65- and 3.53-fold, respectively (*p* < 0.05).

The C_max_ values of NTD were found to be 420.27 ± 66.87, 792.42 ± 55.57, and 1150.61 ± 67.88 ng/mL after administration of the oral NTD suspension, intratracheal NTD suspension, and intratracheal NTD-UFSs, respectively. The intratracheal delivery of NTD-UFSs evoked a significantly higher C_max_ value, around 2.74- and 1.45-fold higher than the oral and intratracheal NTD suspensions, respectively. Furthermore, the NTD-UFSs suspension was computed to have a half-life value of 7.86 ± 0.12 h after intratracheal delivery. Contrarily, the T_1/2_ values were 4.84 ± 0.36 and 4.60 ± 0.35 h for the oral and intratracheal administrations of the NTD suspensions, respectively. The elongated T_1/2_ conferred evidence for the continuous and ameliorated absorption of NTD after intratracheal instillation of NTD-UFSs, elucidating the efficacy of the UFSs nanoreservoir in halting NTD breakdown in the lung.

The F_rel_ of NTD was approximately 160.14 and 565.83% from the intratracheal NTD and NTD-UFSs suspensions, respectively, compared to the oral NTD. Table 4 depicts that the intratracheal instillation of NTD, whether administered as a crude suspension or nanosuspension, substantially improved the pharmacokinetic characteristics compared to the oral NTD suspension. This observation proposes that the targeted therapeutic effect of NTD could be effectively accomplished through pulmonary administration. The joint findings of upraised drug plasma levels, accentuated AUC, and extended T_1/2_ highlighted the potential of the intratracheal application of NTD-UFSs suspension for reinforcing NTD bioavailability in Wistar rats. In general, several characteristics, including the biocompatibility and biodegradability of UFSs components, which conferred more remarkable in vivo tolerability for mucosal membranes, might be responsible for the superior pulmonic NTD pharmacokinetics manifested after the intratracheal administration of NTD-UFSs [11].

Overall, the current work outlines the mechanisms for the optimal formulation of NTD-UFSs to improve pulmonary drug delivery. Interestingly, it was naratted that nano-cargo with a particulate size of less than 500 nm could promote medication deposition in pulmonary tissues, mainly through the enhanced diffusional mobility [23,50]. Because of its small size, the NTD-UFSs formulation improved pulmonary delivery and the deposition of NTD. Moreover, the vesicular nano-cargo’s permeation-enhancing aptitude results from the combined influence of ufasomal hydrophilic and hydrophobic moieties might protrude the penetration of the employed nanosystem [51]. Furthermore, the persistent circulation time triggered from NTD loading into UFSs and the blunted hepatic first-pass metabolism associated with the oral administration of NTD alongside the constringed pulmonary enzymatic drug degradation could contribute to the current snowballed NTD pharmacokinetics.

## 3. Materials and Methods

### 3.1. Materials

Nintedanib was supplied by Dideu Industries Group Limited (Shaanxi, China). Chloroform (HPLC grade), cholesterol, Span 60, Tween 80, formic acid, acetonitrile, dialysis membrane (MW cut off: 12 kDa), methanol, carbamazepine, and sodium carboxymethyl cellulose were supplied by Sigma-Aldrich (St. Louis, MO, USA). Sodium chloride, oleic acid, disodium hydrogen phosphate, potassium dihydrogen phosphate, and potassium chloride were procured from El-Nasr Pharmaceutical Chemical Company (Cairo, Egypt). Also, the investigation comprised analytical laboratory-graded materials.

### 3.2. Design of Experiments

In order to optimize NTD-UFSs, BBD design as a three-factor with three levels was employed. Fifteen NTD-UFSs runs were generated using Design-Expert^®^ software (Version 12.0.3.0, Stat Ease Inc., Minneapolis, MN, USA). A literature review and preformulation studies preceded the selection of the independent and dependent variables. The concentrations (mg) of OA (X_1_), Span 60 (X_2_), and cholesterol (X_3_) were the independent factors. Vesicle size (VS: Y_1_), encapsulation efficiency percent (EE%: Y_2_), zeta potential (ZP: Y_3_, absolute value), and cumulative percentage drug release after 24 h (CR%: Y_4_) were selected as dependent responses. Statistical significance for each response was determined using analysis of variance (ANOVA). The levels of independent factors utilized in the NTD-UFSs optimization method are shown in Table 6. R software (Version 4.2.0, 2022) was utilized in order to create 3D response surface diagrams [52,53]. To identify the optimal NTD-UFSs formulation, the numerical point prediction optimization approach based on the desirability function was utilized. Maximizing EE%, CR%, and ZP (absolute value) while minimizing VS were the criteria used to select the optimum formulation [41].

### 3.3. Preparation of NTD-UFSs

The thin-film hydration method was implemented to fabricate NTD-UFSs with minor modifications [23,54,55]. A round-bottom flask was used to dissolve the calculated amounts of NTD (10 mg), Span 60, cholesterol, and OA in a methanol/chloroform combination (1:1, 10 mL). The Stuart rotary evaporator (RE300, Wolf Laboratories, North Yorkshire, UK) was used to evaporate the organic solvents under vacuum for 15 min at 55 °C. Once a thin, dried, transparent film was formed on the flask’s wall, evaporation was ceased. To finally eliminate any organic solvent residues, the film was vacuum-dried in a desiccator for 2 h. Afterwards, 10 mL of phosphate-buffered saline (PBS, pH 7.4) was added to the film to hydrate it. The formation of UFSs was achieved by maintaining the hydration process at 60 °C (1 h, 100 rpm). A Sonix TV immersion sonicator (model ss-series, North Charleston, SC, USA) was used for 10 min in order to reduce the size of the vesicles. The tailored nanosuspensions of NTD-UFSs were stored at 4 °C overnight prior to further characterization.

### 3.4. Characterization and Optimization of NTD-UFSs

#### 3.4.1. VS and ZP Analyses

The VS and ZP of the NTD-UFSs formulations were evaluated using dynamic light-scattering (DLS) Zetasizer equipment (Malvern, UK). Before the evaluation, the NTD-UFSs formulations were sufficiently admixed with double-distilled water. The average data were subsequently recorded at 25 °C [56].

#### 3.4.2. Encapsulation Efficiency of NTD

Following ufasomal suspension separation, the EE% was calculated indirectly by deducting the quantity of free drug in the supernatant from the total drug content [51]. Separation of the components was achieved by spinning the mixture at 4 °C and 14,000 rpm for 1 h in a SIGMA cooling centrifuge (model 3–30 K, Steinheim, Germany). In order to determine the quantity of non-entrapped NTD at 389 nm (maximum absorbance), the supernatant was separated and diluted before measurement with a Shimadzu UV spectrophotometer (model UV-1601 PC, Tokyo, Japan). Prior to the experiment, a calibration curve was established in PBS pH 7.4 within the concentration range of 2–18 μg/mL (R^2^, 0.9971). In order to determine the EE% of NTD, the following formula was used [41]:

(1) $$EE\%=\frac{Intial\;amount\;added-amount\;in\;supernatant}{Intial\;amount\;added\;}\times 100$$

#### 3.4.3. Cumulative Release (CR) After 24 h

The modified vertical Franz diffusion cells with a diffusional area of 5 cm^2^ were employed for in vitro drug release [57]. A dialysis membrane was submerged in PBS pH 7.4 the day prior to the experiment. Different NTD-UFSs formulations, each containing 3 mg of NTD, were placed in the donor chamber. The receptor chamber was filled with 70 mL of PBS solution pH 7.4 containing Tween 80 at a concentration of 0.5% *w*/*v* [46]. The experiment was carried out with constant stirring at a speed of 100 rpm at a temperature of 37 ± 0.5 °C [46]. Over the course of 24 h, samples were collected and analyzed spectrophotometrically for NTD content at a wavelength of 389 nm to measure the amount of NTD that was released.

### 3.5. Characterization of the Optimized Formulation

#### 3.5.1. Fourier-Transform Infrared Spectroscopy (FTIR)

FTIR spectrometer (IR435-U-04, Shimadzu, Kyoto, Japan) was employed to explore the chemical interactions amongst NTD and the functional groups of the components employed in the UFSs synthesis. An inert environment was used to perform the KBr pellet approach in the FTIR spectrometer at 4000–400 cm^−1^ [10,51]. The spectral characteristics of several purified components, such as NTD (pure drug), cholesterol, OA, and Span 60, were analyzed using FTIR spectroscopy. Furthermore, the optimized formulation (NTD-UFSs) was also investigated.

#### 3.5.2. Differential Scanning Calorimetry (DSC)

The use of DSC analysis allowed for the investigation of possible component interactions [10]. A specific quantity ranging from 2 to 6 mg was obtained by weighing each sample, which included NTD (pure drug), cholesterol, OA, Span 60, and the optimized formulation (NTD-UFSs). The weighed samples were then placed in aluminum containers. Afterwards, the aluminum plates were hermetically enclosed. A calorimeter (DSC-60, Shimadzu, Kyoto, Japan) was employed to conduct the DSC investigation. The reference sample was an empty aluminum pan. In the DSC chamber, the pan containing the sample and the reference was heated from 25 to 300 °C. The flow rate of nitrogen gas was maintained at 20 mL/min, while the heating rate remained consistent at approximately 10 °C/min. Under the same experimental conditions, two empty aluminum containers were also used to calibrate the baseline.

#### 3.5.3. In Vitro Release

Following the above-mentioned description, the release of the optimized formulation and crude NTD was executed. The donor chambers were filled with 3 mg of NTD from both the optimal NTD-UFSs formulation and crude NTD. To guarantee a consistent volume, samples (1 mL) of the receptor milieu were collected at predetermined intervals for 24 h and replaced with the same volume of fresh receptor milieu. Spectrophotometric analysis was performed on the withdrawn samples, after being filtered, at a wavelength of 389 nm to measure the cumulative released amount at the programmed intervals. A correlation between the quantity of released drug and time was computed.

#### 3.5.4. Morphological Analysis

Transmission electron microscopy (TEM) was utilized to investigate the morphological features of the optimum NTD-UFSs dispersion at 80 kv (TEM-1400, Jeol, Tokyo, Japan) [41,58]. One drop of the recently developed NTD-UFSs dispersion was deposited onto a carbon-coated grid and negatively stained with a 2% *w*/*v* phosphotungstic acid solution. Then, filter paper was utilized to remove any excess of the stain.

#### 3.5.5. Short Term Stability

To evaluate the physical stability of the optimized NTD-UFSs formulation, it was refrigerated at 4 °C for three months [37,59]. At several intervals covering 0, 30, 60, and 90 days, samples of the optimum NTD-UFSs formulation were taken and examined for their EE%, VS, and ZP.

#### 3.5.6. Aerodynamic Characterization

Anderson Cascade Impactors (ACIs) (Copley Scientific Ltd., Nottingham, UK) are the most-used tools for pulmonologically administered nano-cargos for the determination of the extent and deposition pattern [60]. ACI is an eight-step apparatus that, when applied to the measurement of aerodynamic diameter, can yield useful information on the size of drug-carrying particles in the respiratory system [61]. Larger particles with sufficient inertia adhere to a specific collection plate as the aerosol stream flows, while smaller particles with insufficient inertia are carried to the next impaction stage by the air stream. By analyzing the data from Copley inhalers, we can determine the following: the fine particle dose (FPD), which is the quantity of particles smaller than 5 µm; the fine particle fraction percent (FPF), which is the percentage of the total amount deposited into the throat and stages of the cascade impactor; and the mass median aerodynamic distribution (MMAD), which is the aerodynamic diameter at which half of the aerosolized drug mass is less than the stated diameter.

The particulate droplet size distribution of the emitted drug was detected by ACI following tight insertion to an endotracheal tube (size 5.5) without a cuff at room temperature. To the ACI, 400 µg of NTD-UFSs suspension was discharged. The endotracheal tube was securely threaded into the induction port of the ACI apparatus with an airtight seal on one side, and a syringe holding the medication was attached to the other end. The ACI set was supplied with a vacuum pump and the flow rate was adjusted using an automated digital flow meter to 15 L/min. After that, a specific volume of acetonitrile was used to wash each plate’s contents, and then the medication was tested.

##### Total Emitted Dose

Based on the nominal emitted dose, which reflects the starting amount of medication supplied to the device, the total ejected dosage is the total amount of medication ejected from the mouthpiece [62]. The medicine delivery system was designed to mimic the way the NTD-UFSs suspension would be administered to a rat through an endotracheal tube. The parameters were fine-tuned to mimic the breathing pattern of a healthy rat, and the flow rate was set at 15 L/min at room temperature. Adjacent to the endotracheal tube was a filter holder that contained an electrostatic filter pad. The amount of the overall inhaled dose may be estimated because this filter would capture all aerosols created when the dose was emitted from the syringe. After soaking the filter pads in acetonitrile for 30 s and then subjecting them to sonication for 3 min, the medication that had been deposited on each filter could be removed. Then, to make sure that the delivery mechanism collected the entire dose, a vacuum was applied across the filter [63]. To achieve 6 consecutive measurements, the experiment was repeated using 2 mL of a 200 µg/mL NTD-UFSs suspension that was passed through the endotracheal tube. The endotracheal tube, filters, and tubing were all rinsed with acetonitrile to remove any remaining medication. The quantity of NTD in the samples was estimated using a validated LC-MS/MS technique.

##### Chromatographic Conditions

The LC-MS/MS approach was employed for the quantitative analysis of NTD [64]. The components included a “Zorbax C18 column (4.6 × 50 mm; 3.5 μm PS)”, a “Shimadzu Prominence (Japan) series LC system with degasser (DGU-20A3)”, and an auto-sampler (SIL-20 AC). For chromatographic separation, a gradient elution program was adopted using mobile phase A, which included 0.1% formic acid, and mobile phase B, which was acetonitrile with 0.1% formic acid. The following was the gradient elution program configuration: 0–2 min at 5–20% B, 2–5 min at 20–30% B, 8–12 min at 30–50% B, 12–13 min at 50–50% B, and 13–14 min at 50–5% B. The volume of the sample that was injected was 10 μL, and the rate of flow was 0.40 mL/min. Cadmazepine was the internal standard. A 5 kV ion discharge voltage was set up. The mass spectrometer was set to a positive mode and operated using electrospray ionization (ESI). In the multiple-reaction monitoring mode, NTD was measured using *m*/*z* 540.3 → 113.15, while the internal standard was measured using *m*/*z* 237.1 → 194.25.

### 3.6. In Vivo Experimentations

In this investigation, male Wistar rats of 200–250 g were employed. The rodents were housed in capacious woven wire enclosures, with the provision of unrestricted access to water and food. These enclosures were located within chambers, which were meticulously controlled for humidity, ensuring a consistent temperature of 25 ± 2 °C. Also, the rats were subjected to 12 h of dark and 12 h of light. Ketamine (12.5 mg/kg) and xylazine (1.5 mg/kg) were administered intraperitoneally to induce anesthesia in the animals [23]. An intratracheal Microsprayer^®^ IA-1C instillation device (Penn-Century, Philadelphia, PA, USA) was used to deliver the optimum NTD-UFSs suspension [65]. The animal experimentations were approved via the Animal Ethics Committee of Beni-Suef University (approval code: 024–032) following the principles delineated in the National Institutes of Health Guide for Care and Use of Laboratory Animals. 

#### 3.6.1. Histopathological Examination

A histopathological analysis was performed to observe the alterations in the lung architecture following the administration of the NTD-UFSs suspension. Two equal-sized groups (normal control group and treatment group) of three rats each were utilized. The optimized NTD-UFSs suspension was intratracheally administered for 14 days to the treatment group. The animals were euthanized at the end of the experimentation, and then their lungs were harvested and maintained in 10% formalin for subsequent examination. For the histological assessment, the lung specimens were embedded in paraffin wax blocks and kept for 24 h at 56 °C. Lung sections (5 μm) were subsequently sliced by a micrometer and subjected to light microscopic analysis after being stained with hematoxylin and eosin (H & E) in accordance with the standard procedure that had been previously outlined by Bancroft and Gamble [66]. At different magnifications, photomicrographs of the sections were captured using a light microscope in conjunction with a LEICA digital camera system (DFC290HD, Heerbrugg, Switzerland).

#### 3.6.2. Pharmacokinetic Studies

Sixteen rats were utilized in the pharmacokinetic investigation and randomly assigned into three groups; three animals comprised each group. For both oral and inhalation treatments, the recommendable dose of NTD was 10 mg/kg [67]. Group A received a suspension of NTD in PBS pH 7.4 (2 mg/mL) containing 0.5% *w*/*v* sodium carboxymethyl cellulose. Following intraperitoneal anesthesia, animals in groups B and C were administered 200 μL of NTD suspension and optimized NTD-UFSs, equivalent to 2 mg of NTD, respectively. In order to further clean the syringe and Microsprayer^®^ tubes, 50 μL of a 0.9% saline solution was administered following the administration of intratracheal doses [23]. At the intervals of 0.5, 1, 2, 4, 8, 12, and 24 h, a blood sample of 500 μL was extracted from the rat’s retro-orbital venous plexus. The samples were kept into heparin tubes to prevent coagulation. After obtaining the samples, the plasma was separated via centrifugation, which was run for 15 min at 3000 rpm. Thereafter, 4 mL of acetonitrile was added to the isolated plasma and mixed by vortex for 5 min. The denatured protein that precipitated was subsequently separated using a cooling centrifugation technique that lasted 10 min at 4 °C. The clear layer of supernatant was evaporated with a vacuum concentrator. Then, the resultant residues were injected into the LC-MS/MS apparatus after being reconstituted in the mobile phase, as previously reported.

##### Data Analysis

As add-ins to Microsoft Excel, the PK solver allowed for non-compartmental analysis, which was used to calculate the pharmacokinetic parameters [68]. C_max_, in ng/mL, was the maximal concentration that was recorded to be the highest concentration across the entire experimentation. T_max_ (h), the time needed to reach this maximal concentration, was also calculated. The integration of the plasma concentration–time curve was determined using the trapezoidal rule. Dividing the concentration at the last time point by the elimination rate constant yielded the residual area, which was then used to compute AUC_0–∞_. Hence, the relative bioavailability percentages (F_rel_) of the two intratracheal formulations compared to the oral NTD suspension as a reference standard can be obtained as follows [23]:

(2) $$F_{rel}=\frac{{AUC}_{0–\infty }\;(intratracheal\;formulation)}{{AUC}_{0–\infty }\;(oral\;formulation)\;}\times 100\;\;$$

### 3.7. Statistical Analysis

The experimentations were conducted thrice and listed as the average ± SD. To determine if the differences between the groups were statistically significant, a one-way ANOVA and a Tukey post hoc test were used. Statistical significance was indicated by a *p* value that was less than 0.05.

## 4. Conclusions

In this investigation, a safe nanoformulation of NTD-UFSs was effectively developed to accentuate the plasma bioavailability of NTD. The NTD-UFSs optimal formulation disclosed good encapsulation efficiency and stability levels along with an appropriate nanoscale dimension. Moreover, the NTD-UFSs formulation allowed the drug to be released steadily for longer time intervals. According to the pharmacokinetic findings, the inhalation route is a viable substitute for preserving therapeutic NTD effectiveness, surpassing systemic and local toxicity, and hence, lowering the dosage and clearance issues. Overall, the current findings could highlight the greater therapeutic potential of UFSs as an effective nanovector for the targeted pulmonary administration of NTD.

## Figures and Tables

**Table 1:** Independent factors and observed responses of NTD-UFSs based on Box–Behnken design.

Run	Independent Factors			Dependent Responses				PDI ^$^
	X_1_ (mg)	X_2_ (mg)	X_3_ (mg)	VS (Y_1_, nm)	EE (Y_2_, %)	ZP (Y_3_, mV)	CR (Y_4_, %)	
R1	32.5	50	60	442.86 ± 6.30	65.13 ± 2.32	−35.44 ± 2.96	47.93 ± 2.48	0.382 ± 0.100
R2	50	50	40	547.73 ± 5.12	79.42 ± 3.32	−41.73 ± 2.63	36.03 ± 2.67	0.461 ± 0.056
R3 *	32.5	100	40	395.20 ± 4.95	68.53 ± 2.87	−36.19 ± 1.76	50.26 ± 2.18	0.300 ± 0.095
R4	15	100	20	269.83 ± 5.48	41.04 ± 2.43	−36.43 ± 2.64	84.79 ± 2.17	0.254 ± 0.024
R5	15	100	60	362.60 ± 4.15	50.73 ± 2.55	−31.14 ± 2.18	65.59 ± 3.39	0.214 ± 0.010
R6 *	32.5	100	40	400.23 ± 6.27	69.34 ± 2.57	−36.96 ± 2.38	51.36 ± 2.18	0.211 ± 0.057
R7	32.5	150	60	470.93 ± 4.95	77.59 ± 3.64	−31.92 ± 1.57	44.12 ± 2.86	0.281 ± 0.050
R8	32.5	150	20	392.03 ± 3.08	73.63 ± 4.31	−37.73 ± 1.49	55.67 ± 2.20	0.391 ± 0.012
R9	32.5	50	20	381.20 ± 3.75	57.35 ± 2.16	−39.54 ± 2.26	61.57 ± 3.13	0.215 ± 0.006
R10 *	32.5	100	40	410.80 ± 4.36	70.64 ± 3.71	−36.51 ± 3.25	53.83 ± 3.72	0.339 ± 0.018
R11	15	50	40	296.70 ± 5.89	36.89 ± 2.18	−34.83 ± 3.18	76.37 ± 2.60	0.270 ± 0.091
R12	50	100	60	579.03 ± 5.25	86.81 ± 3.46	−38.32 ± 2.18	28.90 ± 2.36	0.340 ± 0.024
R13	50	100	20	521.17 ± 4.47	80.72 ± 2.38	−43.27 ± 3.43	40.40 ± 3.52	0.335 ± 0.001
R14	50	150	40	572.13 ± 5.42	92.83 ± 4.38	−40.46 ± 4.29	32.08 ± 2.87	0.391 ± 0.024
R15	15	150	40	349.43 ± 2.35	54.38 ± 2.14	−33.28 ± 3.86	71.80 ± 2.46	0.271 ± 0.044

NTD: nintedanib; UFSs: ufasomes; X_1_: oleic acid concentration (mg); X_2_: Span 60 concentration (mg); X_3_: cholesterol concentration (mg); VS: vesicle size; EE%: encapsulation efficiency percent; ZP: zeta potential; CR: cumulative release percent following 24 h; PDI: polydispersity index. Listed data are mean values *±* SD (*n* = 3). ^$^ Not enrolled in the optimization process. * Indicates the center points of the design.

**Table 2:** Results of regression analysis for different NTD-UFSs responses for data fitting to various models employing Design-Expert^®^ software.

Source	VS (Y_1_)		EE (Y_2_)		ZP (Y_3_)		CR (Y_4_)	
	F	*p*	F	*p*	F	*p*	F	*p*
Model	406.92	<0.0001	140.51	<0.0001	9.45	<0.0001	165.67	<0.0001
X_1_: Oleic acid concentration	3216.9	<0.0001	1057.50	<0.0001	50.48	<0.0001	1302.04	<0.0001
X_2_: Span 60 concentration	50.20	<0.0001	153.15	<0.0001	4.32	0.0451	16.67	0.0002
X_3_: Cholesterol concentration	289.67	<0.0001	32.61	<0.0001	27.33	<0.0001	156.61	<0.0001
Lack of Fit	2.53	0.0748	0.6747	0.5739	0.1636	0.9201	0.5253	0.6681
Model	Reduced Quadratic		Reduced Quadratic		Reduced Quadratic		Reduced Quadratic	
R^2^	0.9905		0.9731		0.7084		0.9771	
Adjusted R^2^	0.9881		0.9661		0.6335		0.9712	
Standard deviation	10.03		2.95		2.44		2.73	
%CV	2.35		4.41		6.60		5.12	
Adequate precision	63.8587		38.8797		10.6806		42.087	
Predicted R^2^	0.9842		0.9550		0.5099		0.9712	
$VS=402.08+116.14X_{1}+14.51X_{2}+34.85X_{3}-7.08X_{1}X_{2}-8.97X_{1}X_{3}+4.32X_{2}X_{3}+25.29X_{1}^{2}+14.13X_{2}^{2}+5.54X_{3}^{2}$								
$EE=69.51+19.59X_{1}+7.46X_{2}+3.44X_{3}-1.02X_{1}X_{2}-0.9017X_{1}X_{3}-0.9542X_{2}X_{3}-3.61X_{1}^{2}-0.0111X_{2}^{2}-1.07X_{3}^{2}$								
$ZP=36.57+3.53X_{1}-1.03X_{2}-2.60X_{3}+0.075X_{1}X_{2}-0.1083X_{1}X_{3}-0.4583X_{2}X_{3}+1.07X_{1}^{2}-0.0625X_{2}^{2}-0.3125X_{3}^{2}$								
$CR=51.82-20.14X_{1}-2.28X_{2}-6.99X_{3}-0.1533X_{1}X_{2}+1.92X_{1}X_{3}+0.5192X_{2}X_{3}+2.43X_{1}^{2}-0.1715X_{2}^{2}+0.6785X_{3}^{2}$								

NTD: nintedanib; UFSs: ufasomes; VS: vesicle size; EE: encapsulation efficiency; ZP: zeta potential; CR: cumulative release percent following 24 h; R^2^: determination coefficient; CV: coefficient of variation.

**Table 3:** The composition of the optimum dispersion and its response variables.

	X_1_: OA Concentration (mg)		X_2_: Span 60 Concentration (mg)		X_3_: Cholesterol Concentration (mg)	
**Optimum values**	**28.308**		**129.282**		**20**	
	**VS (nm)**	**EE%**		**ZP (mV)**		**CR (%)**
**Predicted**	356.06	64.95		−37.67		63.18
**Actual**	364.62	62.51		−36.07		65.57
**Prediction error (%) ^£^**	2.35	−3.90		−4.44		3.64

OA: oleic acid; VS: vesicle size; EE%: encapsulation efficiency percent; ZP: zeta potential; CR: cumulative release percent following 24 h. ^£^ (Actual–Predicted)/Actual × 100.

**Table 4:** The aerodynamic characteristics of the optimum NTD-UFSs formulation.

Aerodynamic Parameter	Value
TED (µg)	322.26 ± 7.51
TED as percentage of nominal dose (%)	80.57 ± 1.88
FPD (µg)	269.44 ± 11.64
FPF (%)	83.61 ± 2.37
MMAD (µm)	3.09 ± 0.23

TED: total emitted dose; FPD: fine particle dose; FPF: fine particle fraction; MMAD: mass median aerodynamic diameter. Results are mean value ± SD (n = 6).

**Table 5:** NTD plasma pharmacokinetic parameters following the administration of the oral NTD suspension, intratracheal NTD suspension, and intratracheal NTD-UFSs in Wistar rats.

Pharmacokinetic Parameter	Formulation		
	Oral NTD Suspension	Intratracheal NTD Suspension	Intratracheal NTD-UFSs Suspension
C_max_ (ng/mL)	420.27 ± 66.87	792.42 ± 55.57 ^a^	1150.61 ± 67.88 ^a,b^
T_max_ (h)	3.00 ± 0.00	1.00 ± 0.00 ^a^	4.00 ± 0.00 ^a,b^
K_e_ (1/h)	0.1433 ± 0.0105	0.1506 ± 0.0119	0.0882 ± 0.0014 ^a,b^
T_1/2_ (h)	4.84 ± 0.36	4.60 ± 0.35	7.86 ± 0.12 ^a,b^
AUC_0–∞_ (ng h/mL)	2690.06 ± 388.77	4307.82 ± 159.76 ^a^	15221.19 ± 383.44 ^a,b^
AUC_0–24_ (ng h/mL)	2565.36 ± 410.07	4135.11 ± 128.83 ^a^	12999.99 ± 1357.57 ^a,b^
MRT (h)	7.21 ± 0.44	6.55 ± 0.68	12.83 ± 0.16 ^a,b^
F_rel_	---------	160.14	565.83 ^b^

NTD: nintedanib; UFSs: ufasomes; C_max_: maximal drug concentration in plasma; T_max_: time to reach C_max_; K_e_: elimination rate constant; T_1/2_: terminal half-life; AUC_0–∞_: total area under plasma concentration–time curve; AUC_0–24_: area under plasma concentration–time curve from 0 to 24 h; MRT: mean residence time; F_rel_: relative bioavailability. Data denote mean *±* SD (*n* = 3). Using one-way ANOVA followed by Tukey’s post-hoc test. ^a^ *p* < 0.05 versus oral NTD suspension; ^b^ *p* < 0.05 versus intratracheal NTD suspension.

**Table 6:** Box–Behnken design employed for the optimization of NTD-UFSs formulations.

Variable	Design Level		
	Low (−1)	Medium (0)	High (+1)
Independent factors			
X_1_: OA concentration (mg)	15	32.5	50
X_2_: Span 60 concentration (mg)	50	100	150
X_3_: Cholesterol concentration (mg)	20	40	60
Dependent factors	Constraints		
Y_1_: VS (nm)	Minimize		
Y_2_: EE%	Maximize		
Y_3_: ZP (mV)	Maximize (absolute value)		
Y_4_: CR (%)	Maximize		

NTD: nintedanib; UFSs: ufasomes; OA: oleic acid; ZP: zeta potential; VS: vesicle size; EE%: encapsulation efficiency percent; CR: cumulative release percent following 24 h.

## Data Availability

All data in this work are presented in the article.
